# Hospital Readmissions after Acute Coronary Syndromes

**DOI:** 10.5935/abc.20190136

**Published:** 2019-07

**Authors:** Alfredo J. Mansur

**Affiliations:** Instituto do Coração (InCor), São Paulo, SP - Brazil

**Keywords:** Acute Coronary Syndrome, Myocardial Infarction, Hospitalization, Patient Readmission, Hospitals Private, Mortality

Acute ischemic syndromes (myocardial infarction, unstable angina) may occur *de novo in* previously asymptomatic patients or may occur in the course of chronic diseases. After hospital-based acute care therapy, with or without percutaneous or surgical intervention, patients are discharged and receive advice for long-term therapy. However, some patients develop symptoms again in the short-term course and are readmitted to the hospital due to clinical as well as non-clinical causes and with clinical and other non-clinical consequences.^1^

Hospital readmissions deserve studies in different settings and have been reported in medical literature. In one report, the readmission rate after one year in patients older than 65 years surviving myocardial infarction was 49.9% in 4.767 hospitals in United States of America between 2008 and 2010; the readmission rate, as well as mortality, were higher in the first months after discharge and decreased thereafter over 12 months.^2^ Additional studies compared not-for-profit hospitals (15.7%) with proprietary hospitals (16.6%) regarding readmission rates in the first month of discharge without demonstrating an association of hospital ownership with the success in programs devoted to decrease readmission rate; the results of such interventions were not statistically different between the two types of hospitals.^3^ Socioeconomic and ethnic factors were systemic influences without overt manifestations in a specific hospital.^4^ In many instances, the readmission rate was studied as a surrogate marker of quality of care;^5-7^ the heterogeneity of patients and medical conditions made a clear association with the index admission elusive and not easily predicted.^5^

In this issue, colleagues from Aracaju (Sergipe, Brazil) report the study^8^ of a hospital-based sample of 536 eligible patients with acute coronary syndrome admitted to three private hospitals (where costs are usually paid by the patient or through an insurance group) and one public hospital (supported by the government, either city, state or federal government) to scrutinize variables associated with re-admissions. They observed the readmission rate was high (115/536, 21.4%) and the mortality of the readmitted patients was also high - 7%. Acute coronary syndromes and heart failure were the leading causes of hospital readmission, mainly in patients admitted to private hospital facilities,^8^ probably related to characteristics of the access. The authors recognize that a hospital-based study sample without full follow-up data are limitations that may stimulate following the previous suggestion to remain vigilant^2^ for health deterioration after discharge from in-hospital treatment, emphasizing the importance of continuous medical advice, support, and therapy. A previous study with a small sample (Maceió, AL) in the same region of the city of the current study suggested low adherence of patients to medical therapy as a significant influence on the frequency of readmissions and mortality.^9^

Finally, further studies and interventions are to be stimulated in other cities and different clinical settings, either hospital-based or at community medical facilities, taking into consideration local challenges and opportunities to make therapy more successful by preventing clinical deterioration that need hospital re-admissions after a period of in-hospital treatment of acute ischemic syndromes.
